# Venereal Prevention

**Published:** 1920-07-24

**Authors:** 


*To the Editor of* The Hospital.

### Venereal Prevention.

*To the Editor of* The Hospital.

Sir,—Who was responsible for the shyness of our first parents, when they deemed it advisable to cover themselves with leaves or skins? They also must be largely responsible for the concealment to-day of venereal disease.

No one doubts that the judicious advertisement of the dangers arising from insufficient precaution before or after contact is a step in the right direction. It goes far to pierce the hidebound conventionalism inseparable from Mrs. Grundy. But Press announcements, one feels, should not be written in the style of a popular novel dealing with sex improbabilities; and the point of view that treats as sensationally improper such an everyday happening, say, as "mixed bathing," should be deleted.

Medical men are all too diffident to ask questions of patients whom they may regard as having probably at some time suffered (or may be then suffering) from venereal disorder. The whispered "aside" should lose its secrecy, and would do so, if the patient recognised that the doctor's question was imperative.

The problem of procreation must become a part of the school curriculum in the higher forms, and the broad shaft of publicity allowed to light up the dark places of the mind at an age when it is of paramount importance. Call it "natural history" or whatever one wishes; much can be learnt by young people from the study of plants, insects, and animals. (Probably it was some thought such as the foregoing that prompted "Social Reformer" in your issue of the 17th inst.) He mentions the instance of the issue to soldiers of packets for self-disinfection, but that the men still contracted infection. Since the Armistice, however, the opportunity for contact may have increased, owing to the enormous reduction in the personnel of the Services and the consequent higher proportion of civil population, but the desire should have decreased with the secession from Army life and training. In the ranks of one of our most famous regiments it is a gibe that unless a man has had syphilis at least three times he cannot be considered a good soldier. This view is endorsed by many non-commissioned officers, and the fact of his suffering from venereal disease during his term of service with the Colours—provided he does not conceal his so doing—seems to be winked at by the authorities.

Those who have formed one of the rough crowd that greets the squad instructor, when first he discusses the etiquette of the professional soldier with his new recruits, will not be surprised that the prevention of venereal disease ranks as one of the most important after-war problems; and anyone who has been disciplined to the accompaniment of the free-and-easy use of highly-explosive and expressive adjectives on the barrack square will doubtless not be surprised to learn from Dr. Addison "that the success of the measures used among soldiers was by no means as complete as is commonly reported."—Yours truly,

Ex-Guardsman.

[We have to acknowledge other letters on the above subjects, but, owing to pressure on our space, they are unavoidably held over until next week.—Ed. The Hospital.]

